# Supplementary material for: Integrated Analysis of Long Non-Coding RNA and mRNA Expression Profiles in Testes of Calves and Sexually Mature Wandong Bulls (Bos taurus)
Source: Animals (Basel). 2021 Jul 5;11(7):2006. doi: 10.3390/ani11072006 (PMC8300165; doi:10.3390/ani11072006)
Supplement: Supplementary file 1 [file animals-11-02006-s001.zip › Figure S4.pdf]

.

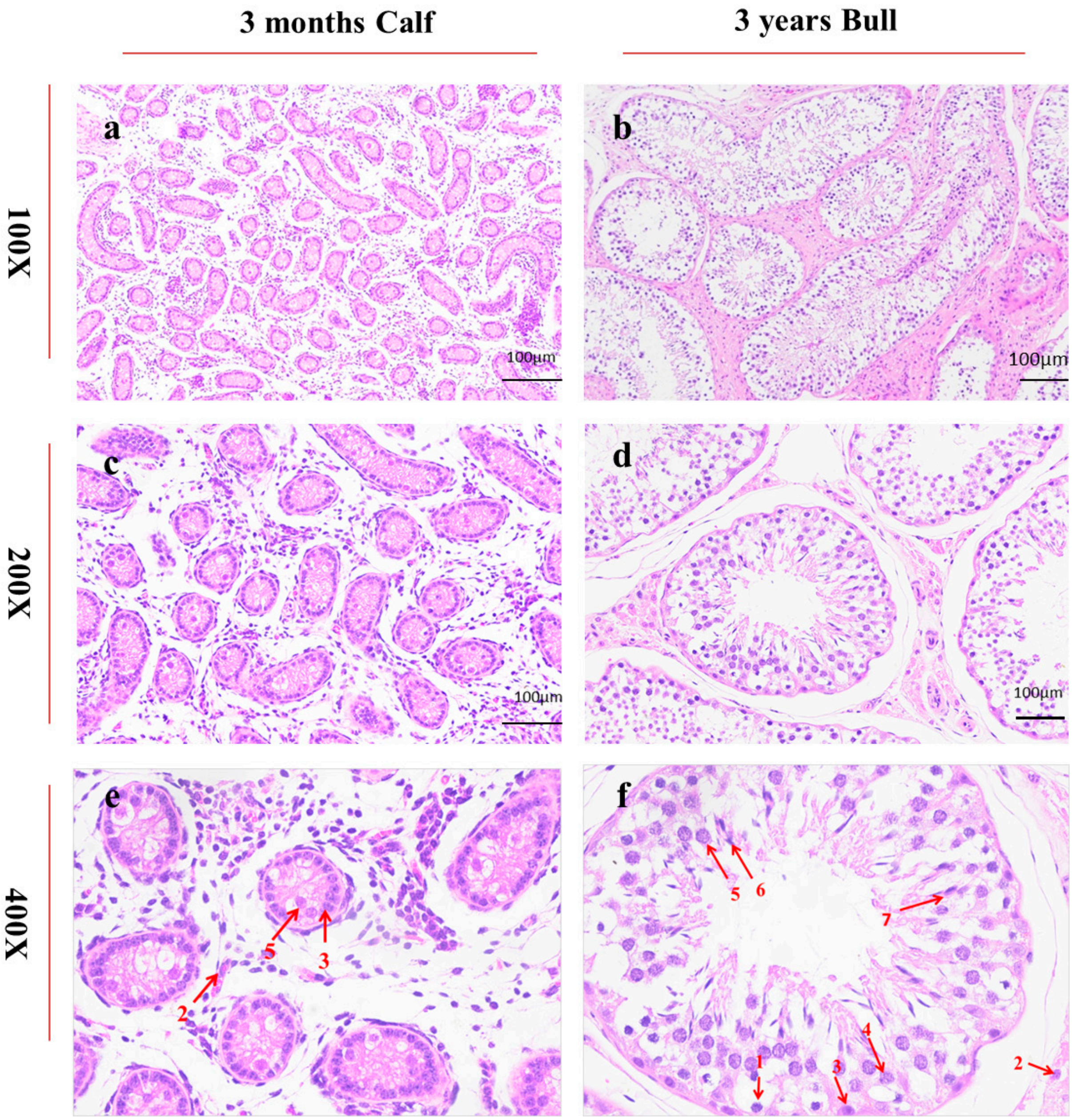

Figure S4: The histomorphological analysis of bull testicular tissues at three months and three years ages were observed under microscope at ×100, ×200, and × 400 magnifications. Bars = 100 μm. The segments (a), (c) and (e) represent the morphology of 3-month-old testes, whereas (b) (d), and (f) represents the morphology of 3 years bulls. The red arrows indicate different cells types. 1: spermatogonia, 2: Leydig cells, 3: Sertoli cells, 4: spermatocytes, 5: round spermatids, 6: elongated spermatids, 7: sperm.
