# Supplementary material for: Integrated Analysis of Long Non-Coding RNA and mRNA Expression Profiles in Testes of Calves and Sexually Mature Wandong Bulls (Bos taurus)
Source: Animals (Basel). 2021 Jul 5;11(7):2006. doi: 10.3390/ani11072006 (PMC8300165; doi:10.3390/ani11072006)
Supplement: Supplementary file 1 [file animals-11-02006-s001.zip › Table S1.pdf]

Table S1. Scrotal circumference, grass motility threshold and progressive motility were evaluated for the sexually mature bulls.

Scrotal circumference		Gross motility		Progressive motility	
Age	Cm	Motility	Scoring	Motility	Scoring
≥15 - ≤18	31	Fast swirling	Excellent	≥70%	Excellent
≥18 - ≤21	32	Slower swirling	Good	50-69%	Good
≥21 - ≤24	33	General oscillation	Fair	30-49%	Fair
>24	34	Sporadic oscillation	Poor	≤30%	Poor
