# Supplementary material for: Integrated Analysis of Long Non-Coding RNA and mRNA Expression Profiles in Testes of Calves and Sexually Mature Wandong Bulls (Bos taurus)
Source: Animals (Basel). 2021 Jul 5;11(7):2006. doi: 10.3390/ani11072006 (PMC8300165; doi:10.3390/ani11072006)
Supplement: Supplementary file 1 [file animals-11-02006-s001.zip › Table S2.pdf]

Table S2: Primers used for RT-qPCR validation of m-RNA and lnc-RNAs sequencing data.

Genes	Accessions no.	Primers sequence (5′→3′)	Product size (bp)
CCDC83	NM_001038218.2	F- AAGAATGTAGGGAGTGAACAGCATGGTA<br>R- TTGTCAATGAACCTTAGAGCATTCTCTG	206
DMRTC2	NM_001038182.2	F-ACATGCTCAACTCTGATACTCCAG<br>R-AGATGAATCTTTCAGTCCCACAAG	163
HSPA2	NM_174344.1	F-CGCTGTGGAGTCCTATACCTATAAC<br>R-CTCTTTCTGCTTGTGTTCATACTCA	175
IQCG	NM_001038195.1	F-ATCCAGTCCATTGGTACATAAAGAA<br>R-TACATCACTCAAAAATTGCCTATCA	169
PACRG	NM_001077027.2	F-AAAGAGACCCTGAGATTAAACAAAT<br>R-CTCTCACGACTGAGTTTTTCATCAT	162
SPO11	NM_001193090.1	F-TCACTCTGGTAGATGCTGATCCACA<br>R-CATCTGGTCCCGTTTTGTCAGTGGA	194
EHHADH	NM_001075780.2	F-TGCCTGCTGCACTTGACTTGATTATCTC<br>R-TGTTGGGCAAGCTCGGGACTGGCATGTT	192
SPP1	NM_174187.2	F-GGCATTGCCTCCGCCCTTCCAGTTAAAC<br>R-CATCAGTTTCCTCAGAGGACACAGAATT	169
NSD2	XM_024993748.1	F-CTGCTCCAAAGGTGGTGAAGTGCGTGAA<br>R-GCCGTTGAACTTCTGCACGACCCCGTCC	170
ACTN4	NM_001098052.1	F-ACCACGCGGCGAACCAGTCGTACCAGTA<br>R-CGGCCTTCCGCAGGTGGGAGTTGCACCA	192
**Genes**	**Accessions no.**	**Primers sequence (5′→3′)**	**Product size (bp)**
COX7A2	NM_175807.1	F-CTCCGTCAGATTGCTAAGAGGACCATAA<br>R-ATGGCATATGCCGTTCCACCAACTGTAA	175
COX6B2	NM_001012684.1	F-AAATGGCCAACGCCGCCTTTCGACCCGC<br>R-GCTGATGGGGCACAGCGAGTGGTACACG	198
PRM2	NM_174157.4	F-GATGCCACGTGAAGAGTCCAACTGAAAG<br>R-CCCTTCTCCGGCGCCTCCGGTAGGGGCG	202
TRIM37	NM_001110182.1	F-AGAGTGTGGAGAGCATTGCTGAGGTGTT<br>R-CAAGCGCGCATCCCGCAGTTTCTCCATG	196
INHBA	NM_174363.2	F-ACATCTTCCCTGTCTCCAGCTGCATCCA<br>R-CCCCTCCTTCTCCATCCCTCTTCTTCCC	165
ERBB4	XM_024981515.1	F-CAAACATTTGTCTACAATCCCACTACCT<br>R-TGCAAGGTTTACACATTTTAATCCCATT	188
SDHA	NM_174178.2	F-TTTCAACACGGCCTGCGTCACGAAGCTC<br>R-GTCCTGGTCCCCCAGCCAGTCGGAGCCT	168
ATP6V0A2	NM_176637.2	F-CCCTGTTCCGGAGCGAGACCATGTGCCT<br>R-GCACCAAGTATGCCAATATTCGCTCCAG	192
FGF9	NM_001245926.1	F-GAACTATTTCGGCGTGCAGGATGCGGTG<br>R-TCCTGCAGTACAGCTGCCTCCGCCTGAG	166
TCF21	NM_001014899.1	F-AGAGATGTGGAGGATCTTCAAGAGGTGG<br>R-AGGGGCTCTTCTTGGTGGGTGCCTTCCT	174
