# Supplementary material for: Integrated Analysis of Long Non-Coding RNA and mRNA Expression Profiles in Testes of Calves and Sexually Mature Wandong Bulls (Bos taurus)
Source: Animals (Basel). 2021 Jul 5;11(7):2006. doi: 10.3390/ani11072006 (PMC8300165; doi:10.3390/ani11072006)
Supplement: Supplementary file 1 [file animals-11-02006-s001.zip › Table S3.pdf]

Table S3: Raw data quality summery of calves and bulls testes sequencing data.

**Sample_name**	**Raw_reads**	**Clean_reads**	**Raw_bases (G)**	**Clean_bases (G)**	**Error rate(%)**	**Q20(%)**	**Q30(%)**	**GC_content(%)**
Bull_1	142165686	140666436	21.32	21.10	0.03	97.40	92.90	52.53
Bull_2	141654378	139622682	21.25	20.94	0.03	97.42	92.94	51.48
Bull_3	117468592	115051574	17.62	17.26	0.03	97.38	92.87	51.98
Calf_1	142605328	140436238	21.39	21.07	0.03	97.50	93.11	53.57
Calf_2	115463518	112484344	17.32	16.87	0.03	97.23	92.64	53.34
Calf_3	118785371	116004150	17.82	17.40	0.03	97.25	92.53	47.09
