# Supplementary material for: Integrated Analysis of Long Non-Coding RNA and mRNA Expression Profiles in Testes of Calves and Sexually Mature Wandong Bulls (Bos taurus)
Source: Animals (Basel). 2021 Jul 5;11(7):2006. doi: 10.3390/ani11072006 (PMC8300165; doi:10.3390/ani11072006)
Supplement: Supplementary file 1 [file animals-11-02006-s001.zip › Table S4.pdf]

Table S4: The top 10 significantly enriched GO description of BP, CC and MF for mRNAs targets genes.

GO ID	GO description	Total gene count	Corrected *p*-*value*
**Biological processes**			
GO:0000003	reproduction	405	8.26E-15
GO:0048610	cellular process involved in reproduction	234	3.51E-13
GO:0048232	male gamete generation	152	3.51E-13
GO:0019953	sexual reproduction	226	3.51E-13
GO:0007283	spermatogenesis	151	5.04E-13
GO:0022414	reproductive process	335	9.37E-13
GO:0032504	multicellular organism reproduction	228	1.53E-11
GO:0007276	gamete generation	185	2.69E-11
GO:0044702	single organism reproductive process	254	3.39E-11
GO:0007286	spermatid development	69	2.47E-10
**Cellular components**			
GO:0097223	sperm part	82	6.93E-17
GO:0001669	acrosomal vesicle	46	8.56E-11
GO:0005930	axoneme	67	9.72E-09
GO:0036126	sperm flagellum	43	2.34E-07
GO:0005929	cilium	168	2.78E-06
GO:0005576	extracellular region	628	8.30E-06
GO:0042995	cell projection	518	1.04E-05
GO:0031514	motile cilium	117	0.00016024
GO:0005780	extrinsic to intraperoxisomal membrane	19	0.0018644
GO:0030312	external encapsulating structure	19	0.0018644
**Molecular function**			

GO:0008827	cytochrome o ubiquinol oxidase activity	43	0.00012968
GO:0005198	structural molecule activity	268	0.00016147
GO:0005179	hormone activity	60	0.0046066
GO:0003777	microtubule motor activity	47	0.0051093
GO:0003916	DNA topoisomerase activity	35	0.0078641
GO:0005215	transporter activity	474	0.011111
GO:0004673	protein histidine kinase activity	14	0.012524
GO:0005201	extracellular matrix structural constituent	33	0.023497
GO:0015077	monovalent inorganic cation transmembrane transporter activity	157	0.023752
GO:0004672	protein kinase activity	237	0.049041
