# Supplementary material for: Integrated Analysis of Long Non-Coding RNA and mRNA Expression Profiles in Testes of Calves and Sexually Mature Wandong Bulls (Bos taurus)
Source: Animals (Basel). 2021 Jul 5;11(7):2006. doi: 10.3390/ani11072006 (PMC8300165; doi:10.3390/ani11072006)
Supplement: Supplementary file 1 [file animals-11-02006-s001.zip › Table S5.pdf]

Table S5. The top 10 most significant enriched pathways regulated by mRNAs in the *bovine* testes development

Terms	Source genes	Total genes	P-value	Corrected P-value
**Adherens junction**	59	68	1.31296794924e-10	3.59753218092e-08
**Cell adhesion molecules (CAMs)**	94	154	7.94775346314e-10	1.08884222445e-07
**Ribosome**	67	136	4.56937630365e-05	0.00417336369066
**ECM-receptor interaction**	47	85	7.76067129299e-05	0.0053160598357
**Amoebiasis**	58	117	0.000125213236461	0.00686168535808
**Lysine degradation**	31	54	0.000756358938021	0.034540391503
**Morphine addiction**	45	93	0.0009887455281	0.038702324957
**Protein digestion and absorption**	41	83	0.00119694229053	0.0409952734505
**Calcium signaling pathway**	76	188	0.00166638615186	0.0507322006233
**Gap junction**	42	89	0.00205415390132	0.0562838168962
