# Supplementary material for: Integrated Analysis of Long Non-Coding RNA and mRNA Expression Profiles in Testes of Calves and Sexually Mature Wandong Bulls (Bos taurus)
Source: Animals (Basel). 2021 Jul 5;11(7):2006. doi: 10.3390/ani11072006 (PMC8300165; doi:10.3390/ani11072006)
Supplement: Supplementary file 1 [file animals-11-02006-s001.zip › Table S6.pdf]

Table S6. The top 10 significantly enriched GO descriptions of BP, CC and MF and regulated the targets genes of lnc-RNAs.

GO ID	GO description	Total gene count	Corrected p-value
**Biological processes**			
**GO:0048518**	positive regulation of biological process	2245	2.31E-31
**GO:0048522**	positive regulation of cellular process	2004	2.11E-29
**GO:0048856**	anatomical structure development	2113	2.66E-29
**GO:0032502**	developmental process	2268	5.73E-26
**GO:0007275**	multicellular organismal development	1910	1.27E-25
**GO:0048731**	system development	1752	1.43E-25
**GO:0032501**	multicellular organismal process	2887	3.43E-24
**GO:0048519**	negative regulation of biological process	1849	2.21E-23
**GO:0051239**	regulation of multicellular organismal process	1226	1.29E-21
**GO:0048869**	cellular developmental process	1616	1.16E-20
**Cellular components**			
**GO:0044464**	cell part	6937	2.38E-61
**GO:0005575**	cellular_component	8177	4.29E-44
**GO:0044424**	intracellular part	5831	7.94E-42
**GO:0005622**	intracellular	5892	5.08E-39
**GO:0043226**	organelle	5098	1.37E-35
**GO:0043229**	intracellular organelle	5081	4.57E-35
**GO:0005737**	cytoplasm	4251	7.33E-35
**GO:0043231**	Intracellular membrane-bounded organelle	4488	6.81E-28
**GO:0044444**	cytoplasmic part	3328	3.22E-22

**GO:0005634**	nucleus	2810	2.97E-17
**Molecular function**			
**GO:0042802**	identical protein binding	661	8.32E-06
**GO:0019899**	enzyme binding	766	3.95E-10
**GO:0044212**	transcription regulatory region DNA binding	425	3.54E-06
**GO:0001067**	regulatory region nucleic acid binding	425	4.09E-06
**GO:0001012**	RNA polymerase II regulatory region DNA binding	346	1.70E-05
**GO:0000976**	transcription regulatory region sequence-specific DNA binding	364	2.04E-05
**GO:0008134**	transcription factor binding	253	3.03E-05
**GO:0000987**	core promoter proximal region sequence-specific DNA binding	232	0.00034545
**GO:0001159**	core promoter proximal region DNA binding	232	0.00034545
**GO:0003700**	sequence-specific DNA binding transcription factor activity	498	0.002096
