# Supplementary material for: Integrated Analysis of Long Non-Coding RNA and mRNA Expression Profiles in Testes of Calves and Sexually Mature Wandong Bulls (Bos taurus)
Source: Animals (Basel). 2021 Jul 5;11(7):2006. doi: 10.3390/ani11072006 (PMC8300165; doi:10.3390/ani11072006)
Supplement: Supplementary file 1 [file animals-11-02006-s001.zip › Table S7.pdf]

Table S7. The top 10 most significant enriched pathways regulated by lncRNAs in the bovine testes development.

Terms	Source genes	Total genes	P-value	Corrected P-value
Ribosome	128	136	3.34531311959e-08	9.23306421006e-06
RNA transport	100	160	0.0210989062546	0.948336203948
Ubiquitin mediated proteolysis	86	138	0.031823016206	0.948336203948
Hippo signaling pathway	93	151	0.0321007421888	0.948336203948
Glycerophospholipid metabolism	59	91	0.0417342824061	0.948336203948
Fructose and mannose metabolism	25	32	0.0450948671249	0.948336203948
TGF-beta signaling pathway	52	79	0.045248655271	0.948336203948
Lysine degradation	37	54	0.0580794954844	0.948336203948
Huntington's disease	112	194	0.062710184272	0.948336203948
Spliceosome	77	130	0.0781095127538	0.948336203948
