# Supplementary figures and images for: Integrated Analysis of Long Non-Coding RNA and mRNA Expression Profiles in Testes of Calves and Sexually Mature Wandong Bulls (Bos taurus)
Source: Animals (Basel). 2021 Jul 5;11(7):2006. doi: 10.3390/ani11072006 (PMC8300165; doi:10.3390/ani11072006)

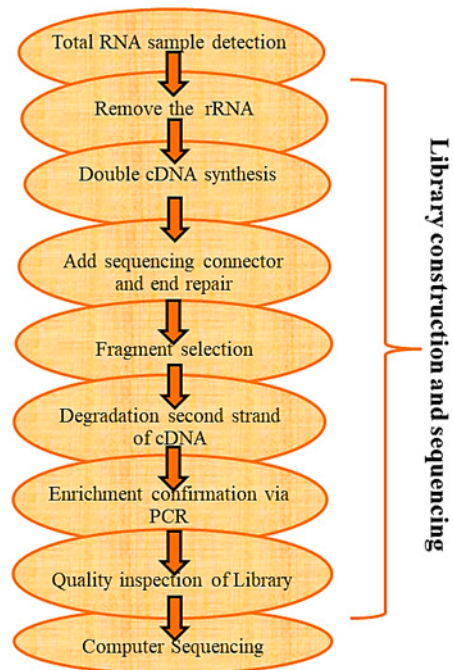

**Figure S2:** Flow chart was adopted for cDNA library construction, and quality inspection.

Supplement: Supplementary file 1 [file animals-11-02006-s001.zip › Figure S2.pdf]

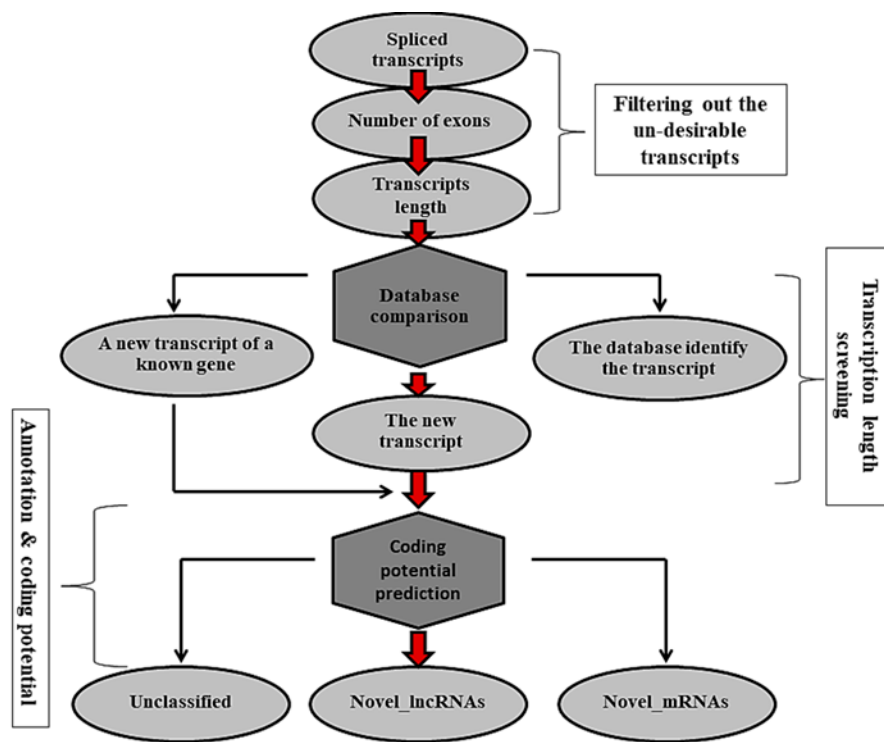

**Figure S3.** The different steps are involved in screening of novel lnc-RNAs and mRNAs.

Supplement: Supplementary file 1 [file animals-11-02006-s001.zip › Figure S3.pdf]
